# Fatty Acid Profile and Desaturase Activities in 7–10-Year-Old Children Attending Primary School in Verona South District: Association between Palmitoleic Acid, SCD-16, Indices of Adiposity, and Blood Pressure

**DOI:** 10.3390/ijms21113899

**Published:** 2020-05-30

**Authors:** Sara Bonafini, Alice Giontella, Angela Tagetti, Irene Bresadola, Rossella Gaudino, Paolo Cavarzere, Diego Alberto Ramaroli, Lorella Branz, Denise Marcon, Angelo Pietrobelli, Pietro Minuz, Franco Antoniazzi, Claudio Maffeis, Cristiano Fava

**Affiliations:** 1 Department of Medicine, University of Verona, Piazzale LA Scuro 10, 37134 Verona, Italy; alice.giontella@gmail.com (A.G.); angela.tagetti@libero.it (A.T.); denise.m@hotmail.it (D.M.); pietro.minuz@univr.it (P.M.); cristiano.fava@univr.it (C.F.); 2 Department of Surgery, Dentistry, Paediatrics and Gynaecology, University of Verona, Piazzale LA Scuro 10, 37134 Verona, Italy; ire.bre@hotmail.it (I.B.); rossella.gaudino@univr.it (R.G.); paolo.cavarzere@ospedaleuniverona.it (P.C.); diegoalberto.ramaroli@univr.it (D.A.R.); lorellabranz@yahoo.com (L.B.); angelo.pietrobelli@univr.it (A.P.); franco.antoniazzi@univr.it (F.A.); claudio.maffeis@univr.it (C.M.); 3 Pennington Biomedical Research Center, 6400 Perkins Rd, Baton Rouge, LA 70808, USA

**Keywords:** palmitoleic acid, SCD-16, obesity, children, desaturase activity, blood pressure

## Abstract

In previous studies, dietary and circulating fatty acids (FA) and desaturases activity (delta-5 desaturase [D5D], delta-6 desaturase [D6D], and stearoyl-CoA desaturase [SCD-16]) involved in their metabolism were associated with metabolic and cardiovascular disorders. The aim of the study was to assess the association between different FAs and desaturases activity (estimated as product:precursor ratios) with individual cardiovascular risk factors (in particular, anthropometric measurements and blood pressure [BP]) in children. The FA profile was determined on a whole-blood drop in 243 children (age: 8.6 ± 0.72 years) participating in a school-based cross-sectional study. Docosahexaenoic acid (DHA) inversely correlated with indices of adiposity, glucose, and triglycerides. Palmitoleic acid and SCD-16 were directly associated with markers of adiposity and BP, even after adjustment for main confounders. D6D correlated directly with the waist/height ratio. Children with excess weight (>85th percentile; that is overweight plus obese ones) showed higher palmitic acid, palmitoleic acid, and higher SCD-16 activity as compared to normal-weight children. Most of the associations were confirmed in the excess-weight group. Omega-3 FAs, particularly DHA, but not omega-6 FA, showed a potentially beneficial association with metabolic parameters, whereas palmitoleic acid and SCD-16 showed a potentially harmful association with indices of adiposity and BP, especially in obese children.

## 1. Introduction

The quality of fatty acids introduced by diet can influence the development of excess weight and other cardiovascular risk factors. Previous findings and guidelines [1,2], and also recent work from our group in a sample of Caucasian obese children, found a harmful association of saturated fatty acids (FA), in particular palmitic acid (PA), with several cardiovascular risk factors characterizing Metabolic Syndrome (MetS) and non-alcoholic fatty liver disease (NAFLD) [3]. In our previous sample of obese children, omega-6 FA, especially arachidonic acid, were inversely associated with the features of the MetS and NAFLD, suggesting a possible protective effect. Anyhow, the role of omega-6 FA, either total and individual, remains an open field of discussion, and their clinically relevant effects are not completely understood [4]. Indeed, several studies and meta-analyses support a protective effect of omega-3 FA with respect to several cardiometabolic disorders, like weight excess, insulin resistance, blood pressure (BP), and plasma lipid profiles [5,6]. Besides, also the activity of many enzymes involved in FA metabolism can play a role in cardiovascular disease. In particular, a lower activity of delta-5 desaturase (D5D), and a higher activity of delta-6 (D6D) and delta-9 desaturase (SCD-1) have been associated with a poorer cardiometabolic profile in several populations [7,8].

Thus, we aimed at investigating the FA profile and desaturases activity in a sample of children derived from a school-based survey, and to assess their association with individual cardiovascular risk factors, especially obesity and high blood pressure (BP). In particular, our main hypothesis was the existence of a possible harmful association between saturated FA, especially palmitic acid, or D6D and cardiovascular risk factors, in contrast to a possible beneficial association of D5D and polyunsaturated FA, in particular arachidonic acid.

## 2. Results

### 2.1. General Characteristics

The results about the prevalence of overweight, obesity, high BP, other anthropometric and metabolic phenotypes, and dietary habits derived from the Food Frequency Questionnaire (FFQ) in the whole sample are presented in a previous report [9].

Briefly, of the 413 children eligible, we enrolled 309 children (participation rate: 74.8%), 155 were females (50.2%) and 154 males (49.8%), aged 7 to 10 years old (mean ± standard deviation [SD ]: 8.6 years ± 0.72); 40 children (12.9%) were obese (BMI ≥ 95th percentile for sex and age), and 65 (21%) were overweight (body mass index [BMI]: 85–95th percentile for sex and age). None of them were taking cholesterol-lowering therapy.

Fatty acid profile, along with other variables needed for the current report, were available in 243 children (F: 124, 50.8%; obese n = 33, 13.6%; overweight n = 55, 22.6%; general characteristics are presented in Table 1).

Blood drop sampling was missing in 66 subjects because of the lack of consent by the parents or refusal by the child (see participants flow-chart in Supplementary Figure S1). Omega-3 Index, a marker of dietary intake of eicosapentaenoic acid (EPA) and docosahexaenoic acid (DHA) in the preceding months, was low (3.9 ± 0.88%). The sum of all measured omega-3 polyunsaturated fatty acids (PUFA) accounted for 4.2 ± 1.01% of total FA. Omega-6 PUFA were the 37.0 ± 2.7% of total FA in blood drop, and the principal components were linoleic acid (19.9 ± 2.2%) and arachidonic acid (12.2 ± 1.7%). Within saturated FA (37.2 ± 2.8%), palmitic acid was the most abundant (23.0 ± 1.3%), whereas trans-FA were 0.66 ± 0.31%. Oleic acid (OA) represented the 19.3 ± 2.0%, and palmitoleic acid, an omega-7 FA, was 0.76 ± 0.28% of the total whole blood FA.

**Figure 1:**
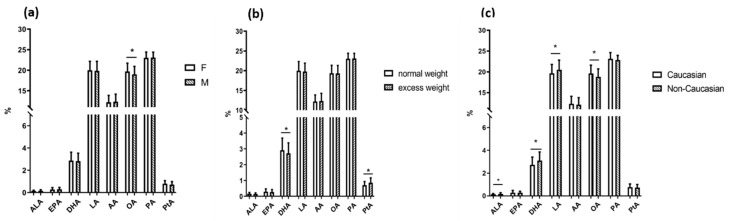
Fatty acid profile of children grouped according to gender (**a**), adiposity (**b**), and ethnicity (**c**). Legend: ALA: alpha-linolenic acid; EPA: eicosapentaenoic acid; DHA: docosahexaenoic acid; LA: linoleic acid;AA: arachidonic acid; OA: oleic acid; PA: palmitic acid; Pta: palmitoleic acid.

Fatty acid profiles of the children grouped according to gender, adiposity, and ethnicity are represented in Figure 1.

### 2.2. Correlations of Fatty Acids and Desaturase Activities with Anthropometric and Clinical Parameters in the Whole Sample

**Figure 2:**
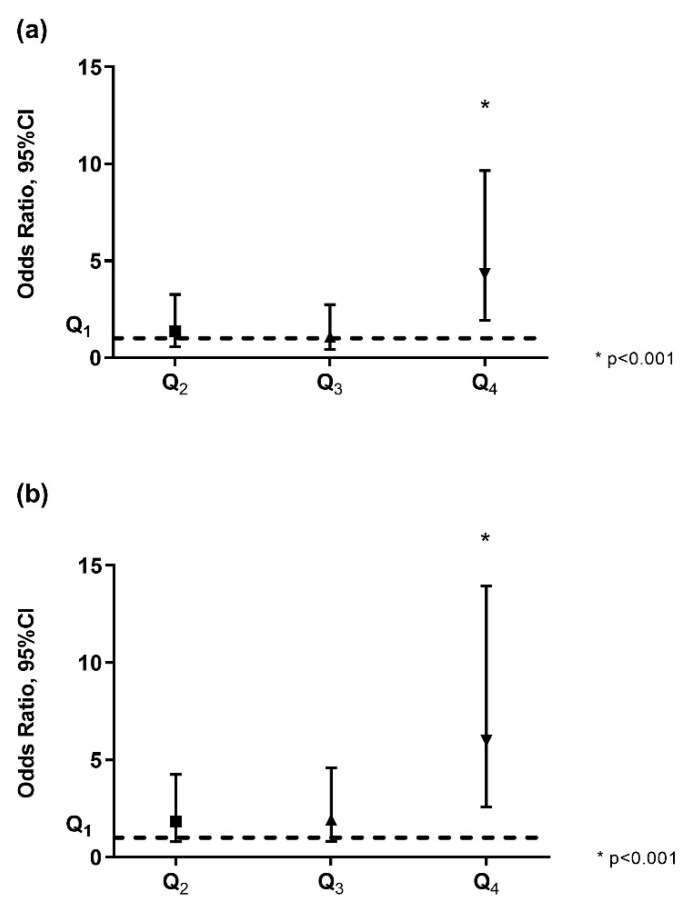
Association of quartiles of palmitoleic acid and SCD-16 to overweight/obesity. Association of quartiles of palmitoleic acid (**a**) and SCD-16 (**b**) to overweight/obesity. Legend: CI: confidence interval; Q1: 1st quartile; Q2; 2nd quartile; Q3: 3rd quartile; Q4: 4th quartile.

Within omega-6 FA, linoleic acid and arachidonic acid did not show significant correlations with anthropometric and hemodynamic features, except for a weak direct correlation of linoleic acid with total cholesterol and triglycerides. Within the omega-3 FA family, DHA was inversely related to BMI, waist/height ratio, capillary glucose, and triglycerides. Palmitic acid, the main saturated FA, was not associated with any of the anthropometric or hemodynamic characteristics. On the contrary, palmitoleic acid showed several highly significant correlations with anthropometric features (BMI: r = 0.408, *p* = 3.5 × 10^−11^; waist/height ratio, fat mass: r = 0.402, *p* = 3.35 × 10^−10^, fat-free mass) and with systolic (SBP) and diastolic BP (DBP). Even the estimated activity of stearoyl-CoA desaturase -16 (SCD-16), the enzyme that metabolizes palmitic acid to palmitoleic acid, was significantly associated with anthropometric characteristics (BMI: r = 0.404, *p* = 6.03 × 10^−11^, waist/height ratio, fat mass: r = 0.409, *p* = 1.6 × 10^−10^; fat-free mass), BP (either SBP and DBP) and triglycerides. Whereas D6D directly correlated to waist/height ratio, glucose and total cholesterol, D5D was not associated with adiposity indices or BP. The correlations of palmitoleic acid and SCD-16 remained significant also after adjustment for main confounders (sex, age, ethnicity, and carbohydrate intake) and multiple testing. Furthermore, the association between SCD-16 and DBP remained significant even when including BMI within the adjustment (Table 2 and Table 3). The 4th quartile of palmitoleic acid level (0.9%–2.1%) and SCD-16 (0.04%–0.08%) were associated respectively with a four-times and six-times-higher odds of association with overweight/obesity as compared to the 1st quartile (odds ratio [OR], 95% confidence interval [CI]: 4.3, 1.9–9.7, *p* < 0.001 for palmitoleic acid; OR, 95% CI: 6.0, 2.6–14.0, *p* < 0.001 for SCD-16) (Figure 2).

### 2.3. Explorative Analysis in Children with Excess Weight

Children with excess weight (overweight + obese subgroups) had higher concentrations of palmitoleic acid, and the estimated SCD-16 activity as compared to normal-weight children (Figure 1 and Table 1). Four children were classified as underweight and were included in the normal-weight group. The excess weight subgroup also showed lower levels of DHA and Omega-3 Index and higher levels of dihomo-gamma-linoleic acid (DGLA) than normal-weight children. In the excess weight subgroup, palmitoleic acid and the estimated SCD-16 activity confirmed their direct correlations with the anthropometric measurements and with either SBP and DBP, also after adjustment for main confounders and multiple testing. Even in the normal-weight group, palmitoleic acid and SCD-16 were associated with anthropometric characteristics but these associations were weaker than in the excess weight group. Moreover, in the overweight and obese children, PA directly correlated with indices of adiposity, even after adjustment for main confounders (sex, age, and ethnicity) (see Table 4).

### 2.4. Explorative Analysis in the Subgroup of Caucasian Children with Excess Weight

We repeated the analysis in the subgroup of Caucasian children with weight excess (n = 55) in order to further investigate the role of PA and of the estimated D6D activity and to test the possible replication of the results of our previous study, conducted in a sample of Caucasian overweight and obese children [3]. In this subgroup, in line with our previous findings, palmitic acid and D6D showed direct correlations with most anthropometric indices and BP (Table 5).

## 3. Discussion

The main hypothesis of this observational study was that polyunsaturated fatty acids could be beneficially associated with cardiovascular risk factors in contrast to a harmful association of saturated fatty acids. In particular, we previously observed that palmitic acid, a saturated FA, and the estimated D6D activity were harmfully associated with several components of the Metabolic Syndrome, whereas arachidonic acid, an omega-6 polyunsaturated FA, and the estimated D5D activity were beneficially associated [3]. Our results highlight that 7–10-year-old students have low levels of omega-3 FA, reflecting scanty dietary intake. In fact, the Omega-3 Index, a well-accepted marker of EPA and DHA consumption, was nearby 4%, far below the suggested protective value of 8% or higher [10]. Moreover, excess-weight children have even lower levels of the Omega-3 Index and DHA compared to normal-weight children. Omega-3 FA are considered beneficial substances for the cardiovascular system and were often associated with lower BP and adiposity [5,6], and in our sample, we find that DHA, the prevalent omega-3 FA, inversely correlated with indices of adiposity, glucose, and triglycerides. This finding could reflect a protective effect of omega-3 FA among several cardiovascular (CV) risk factors, in line with previous reports [11], although the association with the indices of adiposity could just reflect the link among diet and body weight. Anyway, the low levels of omega-3 FA in our sample could have blurred broader protective effects.

Saturated FA are generally considered as unhealthy, particularly for cardiovascular disorders, and current guidelines suggest to limit their intake [12]. Palmitic acid is the most abundant saturated FA, even in children, and it has been associated with obesity and to insulin resistance in humans [13]. In the present study, the association of PA and D6D with clinical variables was not evident in the whole sample but in single subgroups of overweight/obese other than in Caucasian children, suggesting a possible influence of both excess-weight and ethnicity. The influence of ethnicity has already been proposed, although only in a few studies [14]. Even genetic variations in fatty acid desaturase genes (FADS), the genes encoding desaturases, have been tested, identifying several single nucleotide polymorphisms of FADS1 and FADS2 associated with different FA composition and with several CV risk factors [15].

The cardiovascular effects of omega-6 FA are still controversial, with some studies and meta-analysis indicating a protective effect of linoleic acid (LA), and possibly of arachidonic acid (AA), on incident CV disease [4] and other studies reporting no or a possible harmful association of omega-6 FA with CV events [16,17]. Our previous study was in line with the hypothesis of a possible beneficial effect of arachidonic acid on CV risk factors and non-alcoholic fatty liver disease [3]. In this study, arachidonic acid was not associated with vascular or metabolic parameters both in the whole sample and in subgroups. It is worth noticing the different specimens (whole blood drop vs. erythrocytes membrane) used in the two studies. Indeed, the assessment of fatty acids in the blood drop, as in the present study, pool together erythrocyte membrane and plasma FA, thus reflecting both dietary intake and endogenous metabolism. Moreover, plasma measurement is influenced by fasting, especially for fatty acids other than omega-3. Thus, various FA measurements, and the differences in the two samples (different grade of adiposity, different range of age, and pubertal status) could explain, at least partially, the different results.

Interestingly we found an association between palmitoleic acid and estimated SCD-16 activity and some clinical parameters associated with metabolic syndrome. In particular, palmitoleic acid and the estimated SCD-16 activity were associated with markers of adiposity and BP, especially in the excess-weight group. Overweight/obese showed higher levels of palmitoleic acid and estimated SCD-16 activity and stronger associations with adiposity and BP than normal-weight children. Palmitoleic acid, an omega-7 monounsaturated FA (MUFA), is metabolized by the SCD-16 deriving from palmitic acid, a saturated FA, in a process called de novo lipogenesis, whereas the dietary consumption is almost irrelevant. Palmitoleic acid can be metabolized in different tissues, like adipose tissue and liver, with a complex range of metabolic actions and involvement in different metabolic pathways. In experimental models, palmitoleic acid has been favorably associated with some metabolic pathways, whereas several studies in humans reported a potentially harmful association of palmitoleic acid with several cardiovascular risk factors, including BP, insulin resistance, and obesity, even in children [18,19,20]. It has been suggested that several independent factors—such as physical activity and carbohydrate consumption—could influence the endogenous lipogenesis and that different sources of palmitoleic acid, i.e., adipose vs. hepatic tissue, may be related to partially different clinical effects [21,22,23]. Our data support the hypothesis of negative effects of palmitoleic acid on metabolic parameters and BP, which remained significant even after adjustment for important confounders, such as carbohydrate intake.

Stearoyl-CoA desaturase-1 (SCD-1) is the enzyme converting saturated FA palmitic acid and stearic acid into monounsaturated FA—palmitoleic acid and oleic acid, respectively—and is the rate-limiting enzyme in this metabolic chain. In the present work, SCD-1 is split into SCD-16 and SCD-18 to better specify the two pathways starting from palmitic acid (C16:0) and stearic acid (C18:0), respectively. SCD-1 was supposed to protect against the harmful effects of saturated FA accumulation in tissues and bloodstream by metabolizing them into monounsaturated FA [24]. Anyhow, animal models showed an unfavorable association with cardiovascular risk factors [25,26] Although the role of SCD-1 in humans is not completely understood, several studies reported its unfavorable association with cardiometabolic profile, in particular with BP [27], obesity [28] and insulin sensitivity [29], even in children [30]. Also, our results show higher SCD-16 activity in the excess-weight group and direct associations among the desaturase activity and several CV risk factors, which were stronger in overweight/obese than in normal-weight children. Interestingly, the association of SCD-16 with BP was maintained after adjustment for BMI, thus suggesting that the actions of palmitoleic acid on BP are independent of weight excess.

Unresolved issues are whether palmitoleic acid has metabolic effects by itself or it just reflects the enzymatic activity of SCD-16 and how much the SCD-1 activity can influence other metabolic pathways, or it is influenced by different metabolic imbalances. The observational design of this study does not allow any conclusion about causative links among the observed associations, and the effects of the individual FA, in particular, palmitoleic acid, and of desaturases need to be confirmed by different studies and also in different and larger populations It is worth mentioning other limits of this study, as the limited sample size. Moreover, the measurement of the FA profile on erythrocyte membranes is considered the gold standard, but we could measure the FA profile only in the blood drop. Thus, the comparison of the results with those of other studies, included our recent work, should take into account the different specimens. Also, different specimens could reflect, at least partially, different metabolic ways. Furthermore, the uncomplete fasting could have influenced FA levels, especially for essential FA. Anyhow several meaningful associations were found especially for palmitoleic acid, in accordance also with previous studies [18,19,20], and our results remained significant also after adjustment for main confounders, including the estimated total energy and carbohydrate intake, and for multiple testing, thus making our results more trustable. In conclusion, omega-3 and omega-6 FA did not show in the present study a clear association with vascular and metabolic factors or huge differences between obese/overweight and normal-weight children. Despite a generally low level, DHA resulted in weakly associated with BMI and waist/height ratio. Interestingly, we found that, in particular, palmitoleic acid and desaturase activities were associated with the cardiometabolic profile in healthy children. Further investigations are needed to confirm these results in other populations and to better understand the biological mechanisms through whom they can act.

## 4. Materials and Methods

### 4.1. Subjects

Children were recruited from the 3rd and 4th classes of three primary schools of Verona (Italy) South district. The inclusion criteria were the following: children of the abovementioned classes who accepted to participate in the study and whose parents gave written informed consent. The exclusion criteria were either the lack of written informed consent by the parents or refusal to participate by the child.

### 4.2. Study Design

The study was conducted according to a cross-sectional observational design and was approved by the Ethical Committee of Verona (CESC n. 375).

### 4.3. Assessment

Children were recruited from April 2016 to February 2017. They were evaluated in the morning from 8.00 a.m. to 1 p.m. Fasting was not required. A validated food frequency questionnaire (FFQ), integrated with specific questions about PUFA intake [31,32], was collected. Children provided information about ethnicity and the relatives’ Country of origin. Then, the participants underwent a physical investigation. Bodyweight, height, and waist and hip circumferences were measured with the patient wearing light clothes. Bodyweight was measured by a calibrated balance and height by a calibrated stadiometer. Body mass index (BMI) was calculated as weight (kg) divided by the square of height (meters); overweight or obesity was defined for BMI ≥ 85th and 95th percentile for sex and age, respectively [33], according to WHO normograms [34]. Waist circumference was measured to the nearest centimeter with a flexible steel tape measure at the midway between the lowest portion of the rib cage and iliac crest while the subjects were standing at the end of gentle expiration [35]. Waist/height ratio (WHtR) was calculated as waist circumference (cm) divided by height (cm). Waist circumference was transformed in z-score and percentile according to normative values [36]. Through a bioelectrical impedance analysis (Tanita MC 780 MA, Tanita Corporation, Tokyo, Japan) body composition was estimated, in particular, fat (%), fat mass (FM, kg), fat-free mass (FFM, kg), total body water (TBW, kg) and the basal metabolic rate (BMR, kJ, and kcal). During the visit, BP was measured by a semiautomatic oscillometric device specifically validated for children (Omron 705 IT, Omron Corporation, Kyoto, Japan), [37], at least three times, 3 min apart with the patient lying supine for at least 10 min before the first measurement in a room with controlled temperature (22–24 °C). The mean of the three BP measurements was transformed into the z-score and percentile, according to normative values and current guidelines [38,39]. The 95th of office BP measurements was used as the cut-off for hypertension, according to current European guidelines [38].

### 4.4. Vascular Test

To measure carotid-femoral pulse wave velocity (PWV) measurement, a cuff was placed around the femoral artery of the patient to capture the femoral waveform, and a tonometer was used to capture the carotid waveform. The distance between the carotid and femoral arteries was measured, and the velocity automatically determined by dividing the distance by the pulse transit time. The relative z-score and percentile were calculated according to reference values [24,25].

### 4.5. Laboratory Measurements

At 12 a.m., after at least 4 h of fasting, a few blood drops, in willing children, were collected by a fingerprick for plasma cholesterol, triglycerides, and glucose measurements, using two point-of-care testing (POCT) instruments (for cholesterol and triglycerides: HPS Multicare-in, Biochemical System International, Arezzo, Italy; for glucose: and Nova Biomedical, Waltham, MA, USA) [40,41]. For fatty acid analysis, a single drop of scavenged whole blood was collected directly to a filter paper (Ahlstrom 226, PerkinElmer, Greenville, SC, USA) that was pretreated with an antioxidant cocktail (Oxystop, OmegaQuant Analytics, LLC, Sioux Falls, SD, USA) to protect unsaturated FAs from oxidation. After collection, cards were delivered immediately to Omegametrix GmbH (Martinsried, Germany) for analysis by capillary gas chromatography as described previously [42,43]. Fatty acid levels are expressed as a weight percent of the total blood fatty acids (composition). The stability of FAs collected and stored in this manner has been previously evaluated, and no sample degradation was detected [44].

### 4.6. Estimation of Δ^9^, Δ^6^, and Δ^5^ Desaturase Activity

We estimated the desaturase activity as the ratio of product to precursor of individual FA as follows: Δ9-desaturase (SCD-1) = C16:1n-7/C16:0 and C18:1 n-9/C18:0 (referred to as SCD-16 and SCD-18, respectively); Δ6-desaturase (D6D) = C18:3n-6/C18:2n-6 and Δ5-desaturase (D5D) = C20:4n-6/C20:3n-6 [8,28].

### 4.7. Food Frequency Questionnaire

A food frequency questionnaire (FFQ), previously validated for children, was administered [31] and explained to the children and their parents on a previous informative day, then compiled at home along with parents and revised at the evaluation day with each child by a dedicated dietician.

Children indicated their usual consumption of 61 items on the FFQ, using a five-point scale (never; 1–2 times a month; 1–3 times weekly; 4–5 times weekly; one a day; more than once daily). Association of diet to diseases needed to be determined through different approaches because the diet is a complex exposure variable [45]. For more details, we refer to the previous report [9]. Data from FFQ were converted into energy intake (kcal/die) and carbohydrate intake (gr/die). Kcal and carbohydrate content for each food of the FFQ have been retrieved from two Italian food composition tables: the former proposed by “Consiglio per la ricerca in agricoltura e l’analisi dell’economia agraria, CREA” (available online at http://nut.entecra.it/646/tabelle_di_composizione_degli_alimenti.html) and the latter provided by the “Food Composition Database for Epidemiological Studies in Italy” by Gagnarella, Salvini and Parpinel (version 1.2015, website: http://www.bda-ieo.it/) and adapted to standard portions as proposed by the Italian Society of Human Nutrition [(SINU), S.I.d.N.U. Iv Revisione dei Livelli di Assunzione di Riferimento di Nutrienti ed Energia per la Popolazione Italiana (Larn). Available online at http://www.sinu.it/html/pag/tabelle_larn_2014_rev.asp] as previously reported [9]. In the present study, the carbohydrate intake, expressed as the percentage of the total energy intake, and the total energy intake were inserted into a linear regression analysis as cofounding variables, because of the influence of these variables on the SCD-16 activity [21].

### 4.8. Statistics

The normal distribution of the variables was checked by visual inspection and by the Shapiro–Wilk Test. Data are presented as the mean ± standard deviation unless otherwise stated. Children with missing data were excluded from the final analysis. Bivariate parametric correlations were estimated by the Pearson coefficient (r). For CV endpoint variables that were highly correlated with one or more fatty acids or enzyme activity, we run linear regression analyses adjusting for age, gender, Ethnicity (Caucasian vs. non-Caucasian), BMI, and percentage of carbohydrate of total energy intake. Differences between groups were analyzed by Student’s *t*-test. A logistic regression analysis was performed to assess the association of quartiles of palmitoleic acid and of SCD-16 with excess weight/obesity. Successively, an exploratory subgroup analysis, was performed in a pre-specified group of interest (overweight/obese, Caucasian). The statistical analysis and graphs were performed using the Statistical Package for Social Sciences software (SPSS/PC for Windows version 21.0, IBM Corporation, Chicago, IL, USA) and GraphPad Prism software (version 7.00 for Windows, GraphPad Software, La Jolla, CA, USA, www.graphpad.com). *P* values < 0.05 were considered statistically significant.

## Figures and Tables

**Table 1:** General characteristics of the population, split by weight and ethnicity.

	Excess Weight (n: 88)	Normal-Weight (n: 155)	*p* Value *	Caucasian (n: 163)	Non-Caucasian (n: 80)	*p* Value *
**Age (years)**	8.6 ± 0.7	8.69 ± 0.72	0.35	8.68 ± 0.64	8.62 ± 0.85	0.51
**BMI (kg/m^2^)**	21.7 ± 2.8	16.3 ± 1.7	<0.001 ^§^	18.1 ± 3.45	18.4 ± 3.33	0.57
**BMI (percentile)**	93.55 ± 3.7	46.9 ± 26.8	<0.001 ^§^	62.5 ± 31.8	66.5 ± 29.5	0.38
**Waist/Height ratio**	0.51 ± 0.77	0.43 ± 0.07	<0.001 ^§^	0.45 ± 0.09	0.46 ±0.08	0.56
**Fat mass (kg)**	12.9 ± 5.36	5 ± 2.29	<0.001 ^§^	7.31 ± 4.97	9.05 ± 5.82	0.02
**FFM (kg)**	29.68 ± 4.11	25.11 ± 4.06	<0.001 ^§^	26.81 ± 4.81	26.67 ± 4.22	0.82
**PWV (m/s)**	4.77 ± 1.04	4.44 ± 0.84	0.01 ^§^	4.53 ± 0.86	4.62 ± 1.07	0.51
**PWV (Percentile)**	50.97 ± 30	43.13 ± 27.87	0.043	43.99 ± 27.66	49.94 ± 30.88	0.13
**SBP (mmHg)**	112.88 ± 8.6	108.59 ± 10	0.001 ^§^	109.57 ± 9.74	111.33 ± 9.64	0.18
**SBP (Percentile)**	80.31 ± 18.25	72.43 ± 21.42	0.004 ^§^	74.21 ± 20.45	77.48 ± 20.98	0.25
**DBP (mmHg)**	67.52 ± 7.64	65.86 ± 7.43	0.09	65.79 ± 7	67.83 ± 8.38	0.04
**DBP (Percentile)**	71.41 ± 19.04	69.26 ± 20.03	0.41	69.3 ± 18.73	71.53 ± 21.47	0.40
**Glucose (mg/dL)**	89.2 ± 9.1	88.2 ± 10.4	0.47	90.5 ± 9.4	84.7 ± 9.9	<0.001 ^§^
**Cholesterol (mg/dL)**	228.5 ± 39.0	232.6 ± 37.3	0.49	235.3 ± 37.3	222.7 ± 37.7	0.03
**Triglycerides mg/dL)**	191.2 ± 80.6	160.3 ± 67.7	0.005 ^§^	170.6 ± 76.6	170.4 ± 68.6	0.98
**ALA (%)**	0.15 ± 0.06	0.16 ± 0.08	0.17	0.15 ± 0.06	0.17 ± 0.1	0.01
**EPA (%)**	0.27 ± 0.15	0.3 ± 0.17	0.27	0.29 ± 0.18	0.28 ± 0.13	0.69
**DHA (%)**	2.72 ± 0.66	2.92 ± 0.76	0.04	2.72 ± 0.69	3.1 ± 0.76	<0.001 ^§^
**Omega-3 Index (%)**	3.84 ± 0.8	4.08 ± 0.92	0.04	3.86 ± 0.86	4.26 ± 0.86	0.001 ^§^
**LA (%)**	19.8 ± 2.11	19.99 ± 2.32	0.52	19.65 ± 2.15	20.48 ± 2.35	0.008 ^§^
**GLA (%)**	0.22 ± 0.11	0.19 ± 0.1	0.10	0.21 ± 0.1	0.18 ± 0.1	0.05
**DGLA (%)**	2.04 ± 0.35	1.91 ± 0.36	0.008	1.98 ± 0.37	1.91 ± 0.35	0.18
**AA (%)**	12.34 ± 1.92	12.21 ± 1.67	0.57	12.34 ± 1.79	12.11 ± 1.7	0.33
**OA (%)**	19.33 ± 2	19.37 ± 2.02	0.96	19.62 ± 2	18.79 ± 1.94	0.002 ^§^
**PA (%)**	23.1 ± 1.31	23.03 ± 1.4	0.69	23.15 ± 1.47	22.86 ± 1.09	0.10
**Palmitoleic acid (%)**	0.85 ± 0.31	0.7 ± 0.23	<0.001 ^§^	0.77 ± 0.28	0.73 ± 0.27	0.25
**D5D**	6.32 ± 1.81	6.66 ± 1.72	0.14	6.51 ± 1.8	6.58 ± 1.67	0.78
**D6D**	0.01 ± 0.01	0.01 ± 0.01	0.15	0.010 ± 0.01	0.009 ± 0.01	0.02
**SCD-16**	0.04 ± 0.01	0.03 ± 0.01	<0.001 ^§^	0.03 ± 0.01	0.03 ± 0.01	0.37
**SCD-18**	0.65 ± 0.18	0.64 ± 0.22	0.93	0.63 ± 0.22	0.67 ± 0.17	0.12

* Unpaired data *t*-test. ^§^ Significant after adjustment for False Discovery Rate. Excess weight children are defined as BMI ≥ 85th percentile for sex and age; normal-weight children for BMI < 85th percentile for sex and age according to World Health Organization (WHO) normograms. BMI: body mass index; FFM: Fat-Free Mass; PWV: Pulse wave velocity; SBP: systolic blood pressure; DBP: diastolic blood pressure; ALA: alpha-linolenic acid; EPA: eicosapentaenoic acid; DHA: docosahexaenoic acid; LA: linoleic acid; GLA: gamma-linolenic acid; DGLA: dihomo-gamma linolenic acid; AA: arachidonic acid; OA: oleic acid; PA: palmitic acid; D5D: delta-5 desaturase; D6D: delta-6 desaturase; SCD-16: stearoyl-CoA desaturase-16; SCD-18: stearoyl-CoA desaturase-18.

**Table 2:** Correlations of fatty acids and desaturases activities with anthropometric and clinical characteristics in the whole population (n: 243).

	BMI (kg/m^2^)	Waist/Height ratio	FM (kg)	FFM (kg)	PWV (m/s)	SBP (mmHg)	DBP (mmHg)	Glucose (mg/dL)	Chol (mg/dL)	Tg (mg/dL)
**ALA (%)**	−0.010	−0.011	−0.017	−0.017	−0.057	−0.104	−0.089	−0.068	0.012	0.003
**EPA (%)**	0.003	−0.003	−0.016	−0.006	0.053	−0.048	−0.050	0.02	0.015	0.071
**DHA (%)**	−0.144 *_a_	−0.141 *_a_	−0.071	0.003	0.050	0.042	0.047	−0.186 **^§^	−0.049	−0.188 **_b_
**Omega-3 Index (%)**	−0.126	−0.124	−0.065	0.001	0.055	0.028	0.031	−0.160 *	−0.039	−0.150 *
**LA (%)**	−0.057	−0.052	−0.054	−0.066	−0.107	0.111	0.030	−0.064	0.188 *_b_	−0.193 **_b_
**GLA (%)**	0.104	0.158 *_b_	0.074	0.009	−0.021	0.031	−0.084	0.150 *	0.220 **_b_	−0.035
**DGLA (%)**	0.135 *_a_	0.118	0.134 *_a_	0.011	−0.092	−0.006	−0.041	0.018	−0.014	−0.079
**AA (%)**	0.006	−0.008	0.036	0.076	−0.023	−0.050	0.067	−0.041	0.106	−0.048
**OA (%)**	0.029	0.053	−0.016	0.007	−0.079	0.080	−0.034	0.027	−0.138	0.045
**PA (%)**	0.082	0.049	0.018	0.073	−0.055	0.098	−0.109	−0.004	−0.122	0.101
**Palmitoleic acid (%)**	0.408 **^§^_a_	0.308 **^§^_a_	0.402 **^§^_a_	0.258 **^§^_a_	0.079	0.203 **^§^_a_	0.167 **_a_	0.063	−0.110	0.199 **^§^
**D5D**	−0.081	−0.075	−0.064	0.026	0.043	−0.030	0.080	−0.049	0.083	0.025
**D6D**	0.114	0.163 *	0.079	0.024	0.008	0.000	−0.084	0.164 *	0.177 *	0.009
**SCD-16**	0.404 **^§^_a_	0.306 **^§^_a_	0.409 **^§^_a_	0.249 **^§^_a_	0.087	0.192 **_a_	0.184 **^§^_b_	0.068	−0.090	0.180 *
**SCD-18**	−0.041	−0.059	−0.011	−0.056	0.116	−0.151 *_a_	0.027	0.055	0.090	0.117

The underlined correlations remained significant after adjustment for main confounders; the variables included in the regression model are indicated by the subscripts, as follows: a (adjustment for age, sex, ethnicity, carbohydrate intake corrected for total energy intake), b (adjustment for age, sex, ethnicity, BMI, carbohydrate intake corrected for total energy intake). Legend: ** Pearson correlation is significant at the 0.01 level (two-tailed); * Pearson correlation is significant at the 0.05 level (two-tailed); ^§^ significant after Benjamini–Hochberg False Discovery Rate (FDR) adjustment; BMI: bodymass index; Chol: total cholesterol; FM: fat mass; FFM: Fat-Free Mass; PWV: Pulse wave velocity: SBP: systolic blood pressure; DBP: diastolic blood pressure; ALA: alpha-linolenic acid; EPA: eicosapentaenoic acid; DHA: docosahexaenoic acid; LA: linoleic acid; GLA: gamma-linolenic acid; DGLA: dihomo-gamma-linolenic acid; AA: arachidonic acid; OA: oleic acid; PA: palmitic acid; D5D: delta-5 desaturase; D6D: delta-6 desaturase; SCD-16: stearoyl-CoA desaturase-16; SCD-18: stearoyl-CoA desaturase-18; Tg: triglycerides.

**Table 3:** Linear regression model for DBP.

DBP (mmHg)					
	Unstandardized coefficient		Standardized coefficient		
	B	Std. Error	Beta	t	Sig.
	74.710	7.558			
**Age (years)**	−1.841	0.674	−0.175	−2.730	0.007
**Sex**	−0.196	0.963	−0.013	−0.203	0.839
**Ethnicity**	1.890	1.041	0.119	1.815	0.071
**BMI (kg/m^2^)**	0.233	0.151	0.108	1.543	0.124
**Carbohydrate/TEI (%)**	−0.191	0.314	−0.040	−0.611	0.542
**SCD-16**	93.898	46.634	0.142	2.013	0.045

Legend: DBP: diastolic blood pressure; BMI: body mass index; TEI: total energy intake; SCD-16: stearoyl-CoA desaturase-16.

**Table 4:** Correlations of fatty acids and estimated desaturase activities with anthropometric and clinical characteristics in excess-weight and normal-weight children.

	BMI (kg/m^2^)	Waist/Height ratio	FM (kg)	FFM (kg)	PWV (m/s)	SBP (mmHg)	DBP (mmHg)	Glucose (mg/dL)	Chol (mg/dL)	Tg (mg/dL)
**Excess weight group (n: 88)**										
**ALA (%)**	0.121	0.207	0.112	0.024	−0.002	−0.018	−0.183	−0.040	0.047	0.118
**EPA (%)**	0.035	0.133	0.007	−0.021	−0.030	−0.181	−0.204	−0.039	−0.051	0.167
**DHA (%)**	−0.126	−0.130	−0.028	−0.151	0.060	−0.115	−0.148	−0.201	0.053	−0.147
**Omega-3 Index (%)**	−0.103	−0.086	−0.023	−0.136	0.047	−0.138	−0.171	−0.184	0.033	−0.097
**LA (%)**	−0.121	0.001	−0.102	−0.157	−0.046	0.170	−0.024	−0.083	0.220	−0.282 *_a_
**GLA (%)**	0.012	0.232 *	0.011	−0.044	0.069	0.085	0.016	0.144	0.236	−0.087
**DGLA (%)**	0.064	0.135	0.107	0.107	−0.012	−0.017	−0.027	0.150	0.150	−0.044
**AA (%)**	−0.061	−0.127	0.048	0.012	−0.024	−0.032	0.038	0.035	0.156	−0.121
**OA (%)**	0.124	0.066	−0.067	−0.053	−0.105	0.081	−0.026	−0.074	−0.146	0.218
**PA (%)**	0.260 *_a_	0.260 *_a_	0.135	0.198	−0.055	0.131	0.052	0.031	−0.150	0.190
**Palmitoleic acid (%)**	0.395 **^§^_a_	0.271 *_a_	0.395 **^§^_a_	0.186	0.073	0.228^*^	0.406 **^§^_b_	0.024	−0.035	0.289 *
**D5D**	−0.082	−0.162	−0.062	−0.069	−0.041	−0.044	0.029	−0.078	−0.007	−0.056
**D6D**	0.060	0.257 *_b_	0.057	−0.005	0.072	0.066	0.046	0.151	0.199	−0.031
**SCD-16**	0.367 **^§^_a_	0.238 *_a_	0.391 **^§^_a_	0.158	0.084	0.217 *	0.412 **^§^_b_	0.027	−0.010	0.262 *_a_
**SCD-18**	−0.146	−0.152	−0.016	0.012	0.120	−0.205	−0.015	0.081	0.007	−0.029
**Normal-weight group (n: 155)**										
**ALA (%)**	0.078	−0.045	0.029	0.025	−0.063	−0.113	−0.037	−0.079	−0.005	−0.006
**EPA (%)**	0.143	−0.015	0.116	0.058	0.129	0.030	0.040	0.048	0.042	0.047
**DHA (%)**	−0.028	−0.070	0.107	0.186 *_b_	0.085	0.152	0.168 *	−0.175*	−0.104	−0.169
**Omega-3 Index (%)**	0.005	−0.064	0.118	0.175 *_b_	0.101	0.139	0.156	−0.144	−0.081	−0.137
**LA (%)**	0.028	−0.059	0.051	0.007	−0.138	0.101	0.065	−0.053	0.171	−0.145
**GLA (%)**	0.068	0.049	0.023	−0.034	−0.122	−0.032	−0.171 *_b_	0.143	0.213 *	−0.039
**DGLA (%)**	−0.043	−0.013	−0.040	−0.169 *	−0.200 *_b_	−0.055	−0.078	−0.053	−0.082	−0.145
**AA (%)**	−0.005	0.043	−0.041	0.102	−0.036	−0.074	0.082	−0.084	0-082	0.001
**OA (%)**	−0.001	0.068	−0.022	0.026	−0.057	0.088	−0.036	0.070	−0.141	−0.052
**PA (%)**	−0.041	−0.084	−0.204 *_b_	−0.012	−0.063	0.079	−0.201 *_b_	−0.021	−0.119	0.056
**Palmitoleic acid (%)**	0.254 **_a_	0.160 *	0.134	0.114	−0.003	0.115	-0.058	0.068	−0.138	0.055
**D5D**	0.056	0.050	0.057	0.159	0.132	0.007	0.129	−0.029	0.132	0.102
**D6D**	0.078	0.067	0.017	−0.019	−0.058	−0.058	−0.171 *_b_	0.161 *	0.172	0.003
**SCD-16**	0.266 **_a_	0.180 *	0.172 *	0.112	0.002	0.101	−0.025	0.073	−0.119	0.039
**SCD-18**	−0.022	−0.032	0.040	−0.081	0.116	−0.137	0.047	0.05	0.121	0.197 *

The underlined correlations remained significant after adjustment for main confounders; the variables included in the regression model are indicated by the subscripts, as follows: a (adjustment for age, sex, ethnicity, carbohydrate intake corrected for total energy intake), b (adjustment for age, sex, ethnicity, BMI, carbohydrate intake corrected for total energy intake). Legend: ** Pearson correlation is significant at the 0.01 level (two-tailed); * Pearson correlation is significant at the 0.05 level (two-tailed); ^§^ significant after Benjamini–Hochberg FDR adjustment; Chol: total cholesterol; FM: fat mass; FFM: Fat-Free Mass; PWV: Pulse wave velocity: SBP: systolic blood pressure; DBP: diastolic blood pressure; ALA: alpha-linolenic acid; EPA: eicosapentaenoic acid; DHA: docosahexaenoic acid; LA: linoleic acid; GLA: gamma-linolenic acid; DGLA: dihomo-gamma-linolenic acid; AA: arachidonic acid; OA: oleic acid; PA: palmitic acid; D5D: delta-5 desaturase; D6D: delta-6 desaturase; SCD-16: stearoyl-CoA desaturase-16; SCD-18: stearoyl-CoA desaturase-18; Tg: triglycerides.

**Table 5:** Correlations of fatty acids and estimated desaturase activities with anthropometric and clinical parameters in Caucasian children (n: 163).

	BMI (kg/m^2^)	Waist/Height ratio	FM (kg)	FFM (kg)	PWV (m/s)	SBP (mmHg)	DBP (mmHg)	Glucose (mg/dL)	Chol (mg/dL)	Tg (mg/dL)
**ALA (%)**	0.072	0.046	0.032	0.030	−0.118	−0.145	−0.138	0.014	0.056	0.213 *_a_
**EPA (%)**	0.010	−0.005	−0.041	−0.009	0.034	−0.056	−0.060	0.036	−0.003	0.02
**DHA (%)**	−0.136	−0.128	−0.127	0.016	0.041	0.047	0.064	−0.079	−0.013	−0.205 *_a_
**Omega-3 Index (%)**	−0.112	−0.108	−0.116	0.012	0.042	0.027	0.041	−0.059	−0.011	−0.166
**LA (%)**	−0.111	−0.109	−0.111	−0.063	−0.074	0.133	0.012	−0.034	0.234 **_a_	−0.195 *
**GLA (%)**	0.189 *_a_	0.235 **^§^_b_	0.181 *_a_	0.043	−0.043	0.076	-0.045	0.152	0.237 **_a_	0.030
**DGLA (%)**	0.149	0.157 *	0.166 *	0.003	−0.165 *_b_	0.078	−0.011	0.001	−0.052	−0.117
**AA (%)**	−0.032	−0.018	0.002	0.055	0.017	−0.040	0.152	0.037	0.183 *	−0.013
**OA (%)**	0.070	0.041	0.058	0.048	−0.120	0.104	−0.040	−0.115	−0.146	−0.021
**PA (%)**	0.162 *_a_	0.136	0.122	0.117	−0.082	0.189 *_b_	−0.033	−0.075	−0.203 *_b_	0.096
**Palmitoleic acid (%)**	0.428 **^§^_a_	0.325 **^§^_a_	0.445 **^§^_a_	0.254 **^§^_a_	0.098	0.239 **^§^_a_	0.203 **_a_	−0.072	−0.086	0.185 *_a_
**D5D**	−0.119	−0.112	−0.106	0.002	0.096	−0.084	0.111	−0.017	0.162	0.077
**D6D**	0.192 *_a_	0.234 **^§^_b_	0.178 *	0.047	−0.007	0.032	−0.049	0.164 *	0.186 *	0.065
**SCD-16**	0.410 **^§^	0.310 **^§^_a_	0.438 **^§^	0.237 **_a_	0.114	0.213 **_a_	0.207 **_a_	−0.065	−0.047	0.162
**SCD-18**	−0.084	−0.075	−0.076	−0.113	0.147	−0.206 **_b_	−0.008	0.121	0.059	0.153

The underlined correlations remained significant after adjustment for main confounders; the variables included in the regression model are indicated by the subscripts, as follows: a (adjustment for age, sex, ethnicity, carbohydrate intake corrected for total energy intake), b (adjustment for age, sex, ethnicity, BMI, carbohydrate intake corrected for total energy intake). Legend: ** Pearson correlation is significant at the 0.01 level (two-tailed); * Pearson correlation is significant at the 0.05 level (two-tailed); ^§^ significant after Benjamini–Hochberg FDR adjustment; FM: fat mass; FFM: Fat Free Mass; PWV: Pulse wave velocity: SBP: systolic blood pressure; DBP: diastolic blood pressure; ALA: alpha-linolenic acid; EPA: eicosapentaenoic acid; DHA: docosahexaenoic acid; LA: linoleic acid; GLA: gamma-linolenic acid; DGLA: dihomo-gamma-linolenic acid; AA: arachidonic acid; OA: oleic acid; PA: palmitic acid; D5D: delta-5 desaturase; D6D: delta-6 desaturase; SCD-16: stearoyl-CoA desaturase-16; SCD-18: stearoyl-CoA desaturase-18; Chol: total cholesterol; Tg: triglycerides.

## Supplementary Materials

Supplementary materials can be found at https://www.mdpi.com/1422-0067/21/11/3899/s1, Figure S1: Participants flow-chart.

## Abbreviations

AA	arachidonic acid
ALA	alpha-linolenic acid
D5D	delta-5 desaturase
D6D	delta-6 desaturase
DBP	diastolic blood pressure
DGLA	dihomo-gamma-linolenic acid
DHA	docosahexaenoic acid
EPA	eicosapentaenoic acid
PWV	Pulse wave velocity
SBP	systolic blood pressure
SCD-16	stearoyl-CoA desaturase-16
SCD-18	stearoyl-CoA desaturase-18
