# Supplementary figures and images for: Fatty Acid Profile and Desaturase Activities in 7–10-Year-Old Children Attending Primary School in Verona South District: Association between Palmitoleic Acid, SCD-16, Indices of Adiposity, and Blood Pressure
Source: Int J Mol Sci. 2020 May 30;21(11):3899. doi: 10.3390/ijms21113899 (PMC7312303; doi:10.3390/ijms21113899)

**Supplementary Figure S1.** Participants flow-chart.

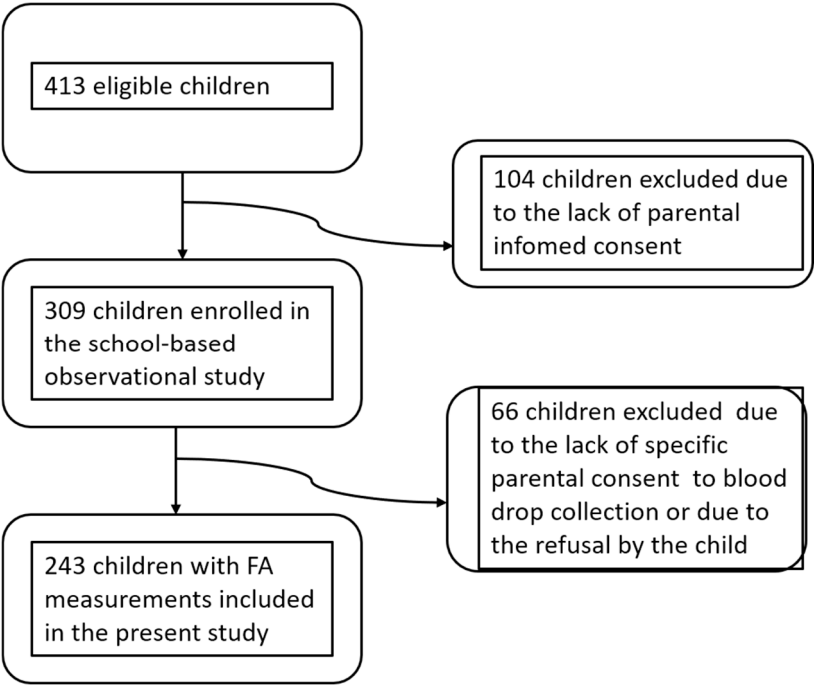

Supplement: Supplementary file 1 [file ijms-21-03899-s001.pdf]
